# Identification and Characterization of the RLIP/RALBP1 Interacting Protein Xreps1 in *Xenopus laevis* Early Development

**DOI:** 10.1371/journal.pone.0033193

**Published:** 2012-03-08

**Authors:** Laurent Boissel, Jonathan Fillatre, Jacques Moreau

**Affiliations:** Institut Jacques Monod, CNRS (UMR7592), Université Paris Diderot, Paris, France; University of Hyderabad, India

## Abstract

**Background:**

The FGF/Ras/Ral/RLIP pathway is required for the gastrulation process during the early development of vertebrates. The Ral Interacting Protein (RLIP also known as RalBP1) interacts with GTP-bound Ral proteins. RLIP/RalBP1 is a modular protein capable of participating in many cellular functions.

**Methodology/Principal Findings:**

To investigate the role of RLIP in early development, a two-hybrid screening using a library of maternal cDNAs of the amphibian *Xenopus laevis* was performed. Xreps1 was isolated as a partner of RLIP/RalBP1 and its function was studied. The mutual interacting domains of Xreps1 and *Xenopus* RLIP (XRLIP) were identified. Xreps1 expressed *in vivo*, or synthesized *in vitro*, interacts with *in vitro* expressed XRLIP. Interestingly, targeting of Xreps1 or the Xreps1-binding domain of XRLIP (XRLIP(469–636)) to the plasma membrane through their fusion to the CAAX sequence induces a hyperpigmentation phenotype of the embryo. This hyperpigmented phenotype induced by XRLIP(469–636)-CAAX can be rescued by co-expression of a deletion mutant of Xreps1 restricted to the RLIP-binding domain (Xreps1(RLIP-BD)) but not by co-expression of a cDNA coding for a longer form of Xreps1.

**Conclusion/Significance:**

We demonstrate here that RLIP/RalBP1, an effector of Ral involved in receptor-mediated endocytosis and in the regulation of actin dynamics during embryonic development, also interacts with Reps1. Although these two proteins are present early during embryonic development, they are active only at the end of gastrulation. Our results suggest that the interaction between RLIP and Reps1 is negatively controlled during the cleavage stage of development, which is characterized by rapid mitosis. Later in development, Reps1 is required for the normal function of the ectodermic cell, and its targeting into the plasma membrane affects the stability of the ectoderm.

## Introduction

During oogenesis, large amounts of RNAs, necessary for early development, accumulate. These stored maternal mRNAs are used to support protein synthesis during the first few hours of development, before the onset of embryonic transcription and up to gastrulation. Among the products of these genes, different components of the fibroblast growth factor (FGF) transduction pathway are present, such as Ras and members of the MAPK family, to ensuring quick responses of the vegetal inducing signals during the mesodermal induction. Previously [1], we characterized, from a subtractive library enriched in maternal mRNA, a new gene encoding a protein that belongs to the FGF/Ras pathway and that is involved in early development [2]. The FGF signaling plays a key role during early development of vertebrates, where it has been implicated in a large number of processes such as induction, patterning of the three germ layer and control of morphogenetic movements. In *Xenopus laevis* embryos FGF signaling was shown to be required for two developmental processes through the involvement of different effectors of Ras: i) mesodermal gene expression through Raf [3] and PI3 kinase [4], and ii) cell movements of gatrulation through the Ral guanine nucleotide dissociation stimulator (RalGDS), which modulates the dynamics of the actin cytoskeleton [5]. RalGDS is a guanine exchange factor (GEF) that can activate the Ral proteins [2], that are members of the superfamily of the Ras small G proteins. Several effectors that bind specifically to the activated form of the Ral proteins have been identified, and they are involved in different cell processes such as protein secretion or endocytosis [6], [7], [8]. The Ral Interacting Protein (RLIP also known as RalBP1) [9] [10], [11] interacts with GTP-bound RalA and RalB. The *Xenopus* RLIP (XRLIP) has been cloned and its primary sequence is 85% identical to the mammalian RLIP76 [12]. During gastrulation of *Xenopus* embryos, XralB [2] and RLIP [12] are involved in modulating the stability of cortical actin assembly. Moreover, RalB requires RLIP for morphogenetic movements at this stage.

**Figure 1:**
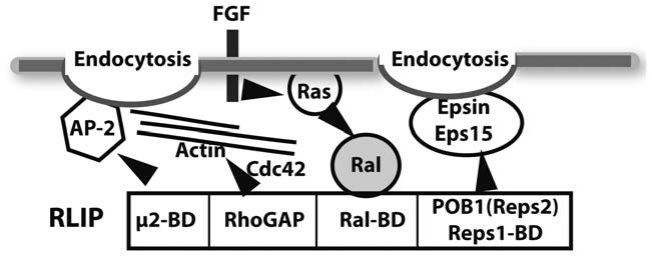
Interacting domain of RLIP involved in endocytosis. RLIP through its N-terminal and C-terminal domains interacts with μ2, and POB1 or Reps1 proteins respectively. These proteins interact in turn with complexes involved in clathrin endocytosis. The RhoGAP sequence of RLIP controls the Cdc42 activity and the actin dynamic. The Ral binding domain binds with Ral, in response to activation tyosine kinase receptor through the activation of the Ras protein.

RLIP/RalBP1 is a modular protein capable of participating in many cell functions (Fig. 1). It contains a RhoGAP domain that enhances the GTPase activity of two members of the subfamily of small G proteins, namely, Rac1 and Cdc42 [10]. These proteins are regulators of the actin cytoskeleton and are also involved in many cell processes, such as membrane trafficking (for a review see [13]). RLIP also binds to the μ2 subunit of the heterotetrameric clathrin adaptor (AP-2 complex) [14] and to the proteins Reps1 [15] and POB1 (also known as Reps2) [16]. Reps1 and POB1/Reps2 share only 38% sequence identity. However, they both contain an Eps15 homology (EH) domain involved in protein-protein interactions, and also polyproline stretches and a coiled-coil domain located in the same order. POB1 interacts directly with Eps15 and Epsin (for reviews see [17], [18]) and Reps1 probably does so as well [19]. Eps15 and Epsin, and their yeast orthologs Pan1 and End3, are essential components of the endocytotic machinery. They can interact with each other and with the clathrin-recruiting AP-2 complex, necessary for endocytosis. Indeed, RLIP and POB1 as well as RalA are involved in the endocytosis of various receptors [20], [14], [21]. RLIP is overexpressed in several tumor cells such as prostate, melanoma, lung and bladder cells, as well as in cancer cell metastasis [22], [23].

We have previously shown that XralB can affect the stability of the F-actin network in *Xenopus* embryos [2] and that it is required for the morphogenetic movements during gastrulation [5]. Similarly to Ral, RLIP induces cortical actin disruption when it is targeted to the plasma membrane to mimic its cellular interaction with Ral [12]. Since RLIP is an effector of Ral present in early development and is required for gastrulation, we have attempted to uncover other proteins involved in this function. Using a two-hybrid screen to identify XRLIP partners in early development, the *Xenopus* ortholog of Reps1 (referred to as Xreps1(253)) was isolated. Whereas Xreps1 and XRLIP interact *in vitro*, this interaction cannot be detected *in vivo* before gastrulation. Interestingly, although Xreps1(253) is presumably not involved in the action of XRLIP during gastrulation, it suppresses the phenotype induced by N-terminally truncated (ΔN-terminal) XRLIP mutants. The lack of phenotypic effect due to overexpression of Xreps1(253) or mouse Reps1 (Mreps1) indicates that this protein is not active before the mid blastula transition (MBT), and that later in development its cellular function could be restricted to ectodermic cells. Indeed, targeting Xreps1(253) or Mreps1 to the plasma membrane causes a persistent hyperpigmentation of ectodermic cells. Since membrane localization of Xreps1(253) relies on its interaction with XRLIP, we suggest that the interaction between these two proteins is required for a function of ectodermic cell after MBT.

## Results

### Characterization of the interaction between XRLIP and Xreps1(253)

**Figure 2:**
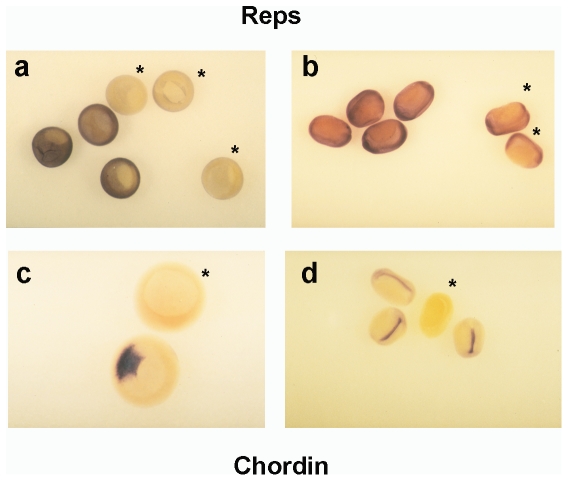
Reps1 is uniformly expressed in early development. *In situ* hybridization of Reps (a and b), and Chordin (c and d) in gastrula (a and c) and neurula (b and d). Each embryo was hybridized with one antisens, and one sens probe. Embryos hybridized with the sens probe are marked with *.

**Figure 3:**
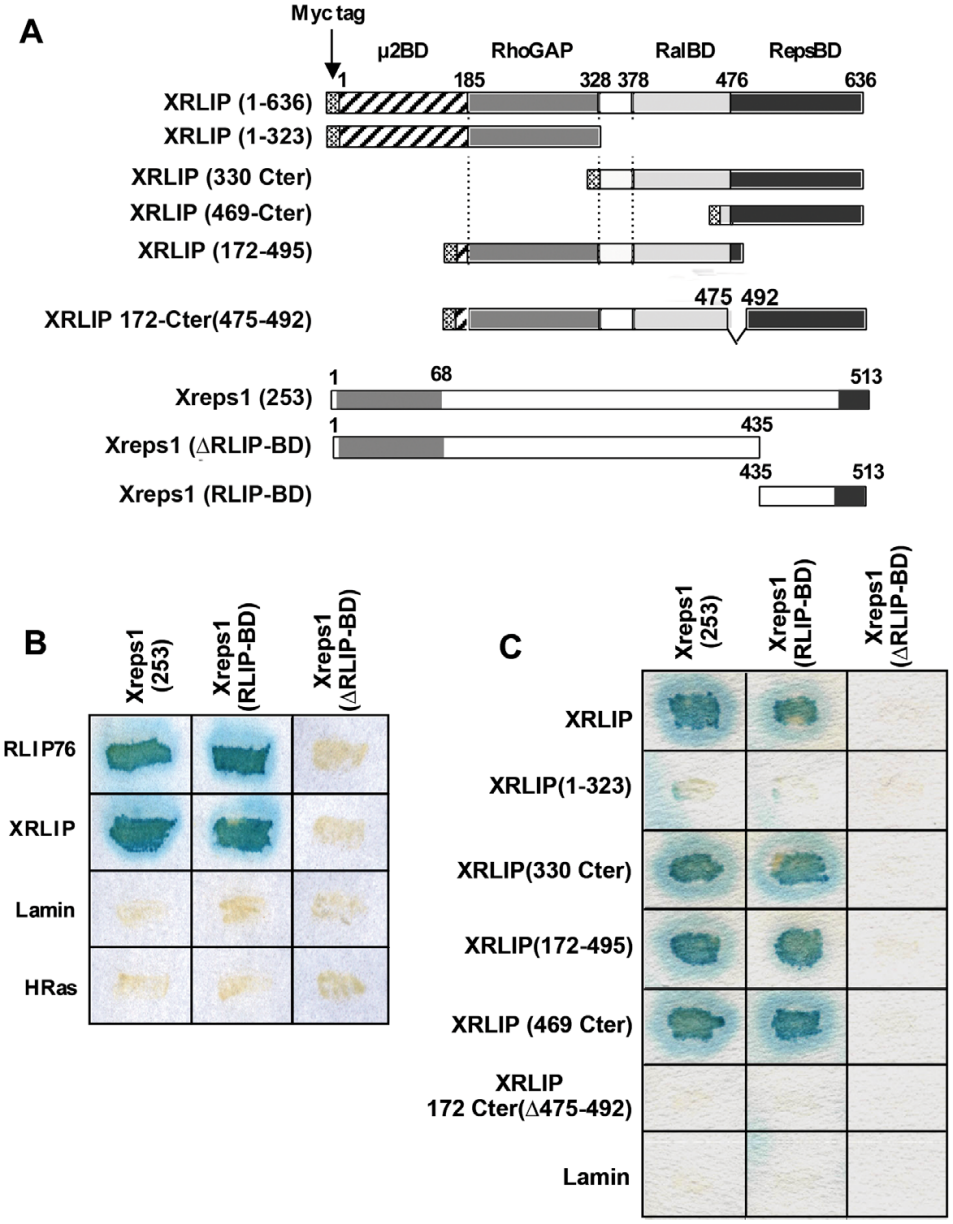
Interaction analysis of XRLIP with Xreps1 by the two-hybrid technique. (A) Schematic representation of the XRLIP and Xreps1(253) deletion mutants used here. For XRLIP, a distinctive box represents each interacting domain. For Xreps1(253), the grey box corresponds to the EH homology domain and the black box to the coiled-coil motif. (B) Two-hybrid assay between Xreps1(253), Xreps1(RLIP-BD) or Xreps1(ΔRLIP-BD) and human RLIP (RLIP76), *Xenopus* RLIP (XRLIP), or Lamin or human Ras (Hras) as controls. (C) Two-hybrid assay between Xreps1(253), Xreps1(RLIP-BD) or Xreps1(ΔRLIP-BD) with different deletion mutants of XRLIP or Lamin as control.

To identify proteins that might participate in early development through binding to RLIP, we screened a *Xenopus* cDNA library constructed at different stages of oogenesis and early development [25] using the yeast two-hybrid system with the full-length human RLIP76 as the bait [14]. Four cDNAs of different lengths coding for Xreps1 were isolated. The longest cDNA (clone 253 referred to as Xreps1(253), Accession number AJ878914 (see below)) codes for a protein of 513 residues that lacks 229 residues from the N-terminal region, compared to the murine orthologous protein. However, this clone that corresponds to a partial coding sequence of the Reps1 gene, encompasses all the domains previously characterized in Reps1, including the potential tyrosine-phosphorylation sites [15]. Sequence comparisons showed 76% of identity between the C-terminal two-thirds of the sequences of Reps1, Mreps1 and Xreps1(253). Identity was higher in the EH domain, rising to 95% for the 49 most C-terminal amino acids. The shortest cDNA fragment codes for a polypeptide of 78 amino acids that overlaps the C-terminal end of Mreps1, including the putative coiled-coil region (residues 690–743 in mice). This clone was named Xreps1(RLIP-BD) (see below). A northern blot study involving RNA samples from stage-VI oocytes to the tadpole stages and from different tissues, probed with Xreps1(253), showed that only one type of mRNA (2.7 kbp) was expressed from oogenesis and throughout development and in the different tissues such as liver, kidney and heart (data not shown). Thus, Reps1 mRNA is clearly present during oogenesis of *Xenopus*, as it is in human ovaries [30], and is detected in the same differentiated tissues tested, such as liver, kidney and heart, as previously reported by Xu et al [30]. We examined whether Reps1 could have a spatial expression profile during early *Xenopus* development by performing *in situ* hybridization using an anti-sens Reps1, and an anti-sens Chordin probe [31] as positive control of the transcript located. Whereas expression of Chordin is clearly detected in the dorsal blastoporal lip (Fig. 2c) or in the notochord (Fig. 2d), Reps1 shows a weak uniform expression (Fig. 2a, b) in the entire embryo. This result confirms the ubiquitary distribution of Reps1 in the embryo as observed by northen blot in adult tissues. The strong sequence identity between Mreps1 and Xreps1(253), as well as the evolutionary conservation of RLIP, prompted us to check whether the interaction between RLIP and Xreps1 was also conserved during amphibian development. First, a yeast two-hybrid approach was used. Specifically, yeast cells were co-transformed with plasmids driving the expression of either human RLIP (known as RLIP76), XRLIP, human Lamin or human wild-type Hras and, on the other hand, with plasmids encoding either Xreps1(253), Xreps1(RLIP-BD) or Xreps1 (ΔRLIP-BD) (Fig. 3A). As shown in Figure 3B, Xreps1(RLIP-BD) interacted with RLIP76 and XRLIP, as did Xreps1(253), whereas Xreps1 (ΔRLIP-BD) failed to bind XRLIP. As expected, Xreps1(253) was unable to interact with Lamin or Hras, excluding potential non-specific interactions in the conditions of the two-hybrid assay. These results showed that i) the interaction between Xreps1(253) and XRLIP was specific, ii) that the domain Xreps1(RLIP-BD) was necessary and sufficient for the interaction to take place and iii) that the interaction Xreps1(253)-XRLIP was conserved from *Xenopus* to humans [15].

The RLIP-binding domain of Reps1 has been mapped between residues 599 and 743 in mice, corresponding to 369 to 513 in the Xreps1(253) clone [15]. The Xreps1 clone called Xreps1(RLIP-BD), and corresponding to the fragment 435–513 of the initial Xreps(253) sequence, retains the ability to interact with XRLIP (Fig. 3B and C). Interestingly, Xreps1(RLIP-BD) is nearly half the length of the previously defined RLIP-binding domain of Reps1 [15]. The identification of such a minimal RLIP-BD in Xreps1 raises the possibility of using this small domain *in vivo* as a negative competitor of its endogenous full-length counterpart.

The Reps1-binding domain of RLIP was previously mapped between residues 500 to 647, which corresponds to positions 481 to 628 of our XRLIP clone. Attempts were made to better define the size of this interacting domain by testing the ability of either Xrep1(253), Xreps1(RLIP-BD) or Xreps1(ΔRLIP-BD) to bind to different deletion mutants of XRLIP in two-hybrid assays. As expected, both Xreps1(253) and Xreps1(RLIP-BD) interacted efficiently with XRLIP(330-Cter) and XRLIP(469-Cter) because these deletion mutants contain the previously described binding region. However, they failed to bind to a truncated version of XRLIP (XRLIP(1–323)) used as control and containing only the μ2 binding domain and the RhoGAP region, and lacking the C-terminal part of the protein. As expected, Xreps1(ΔRLIP-BD) did not interact with any of the XRLIP mutants (Fig. 3C). Interestingly, Xreps1 interacted with an XRLIP deletion mutant containing only the RhoGAP and RalBD (i.e. XRLIP(172–495)). The most C-terminal part of RalBD of XRLIP contains 14 amino acids also present in the Reps-BD, as mentioned above. This suggests that the region of interaction with Reps involves only 14 amino acids located between residues 481 and 495 of XRLIP. To confirm this possibility, we tested in a two-hybrid assay whether XRLIP deleted of the region between amino acids 475 and 492 still had the capacity to bind Xreps1(253). As expected, the construct deleted for this region, XRLIP 172-Cter (Δ475–492), failed to bind to Xreps1(253) (Fig. 3C). This clearly shows that both regions of XRLIP comprised between amino acids 475 and 492, as well as the C-terminal portion of Xreps1 are necessary to mediate the interaction between these two proteins.

### XRLIP and Xreps1(253) interact in vitro

**Figure 4:**
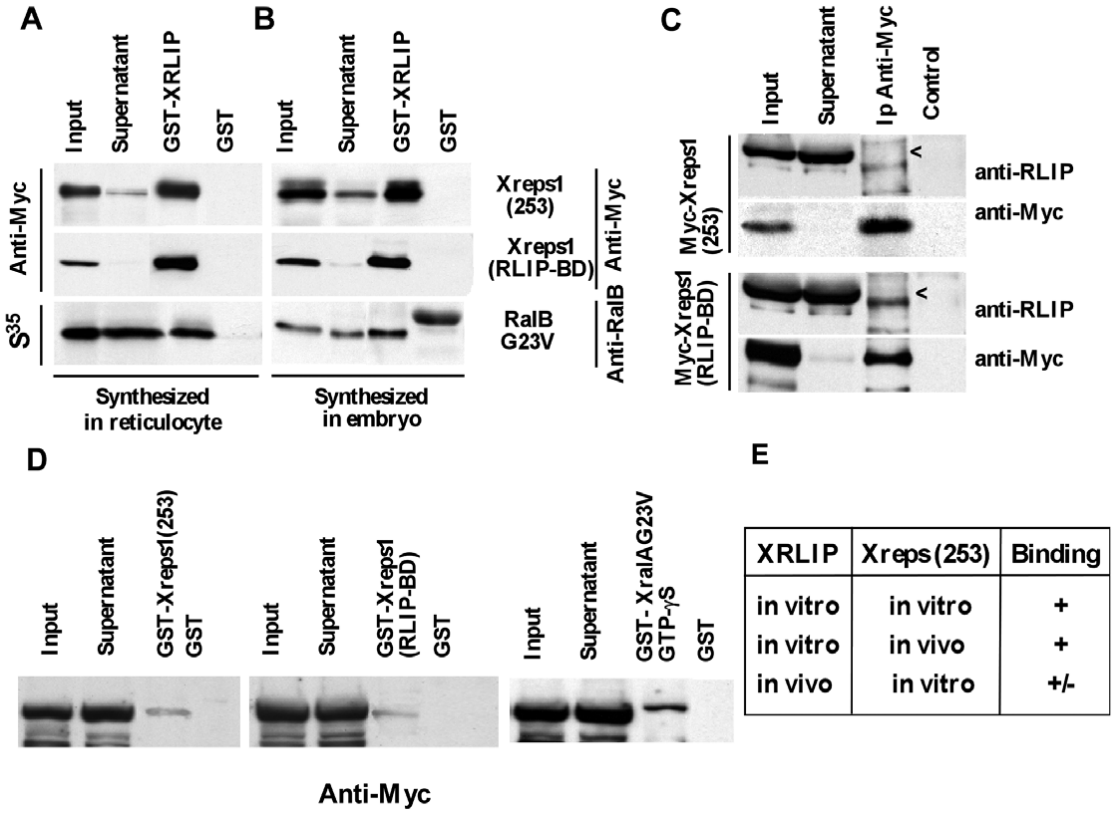
Characterization of the Xreps1(253) domain critical for interaction with XRLIP, and interaction *in vivo* of Xreps1(253) and Xreps1(RLIP-BD) with XRLIP. (A, B) GST-XRLIP or GST alone as control were tested for their ability to pull down Xreps1(253) or Xreps1(RLIP-BD) synthesized as Myc-tagged proteins. The interaction of XRLIP with RalB G23V served as positive control. Xreps1(253) precipitated with 1 µg GST-XRLIP was analyzed. (A) The input or supernatant ranged from 1.25 µl of the 25 µl of reticulocyte extract), or (B) half an embryo from extracts of 10 embryos, were analyzed by SDS-PAGE. (C) Myc-Xreps1(253) or Myc-Xreps1(RLIP-BD) were synthesized in embryos and used to interact with endogenous XRLIP. Total lysate from 10 embryos were incubated with an anti-Myc agarose-conjugated antibody (SC-40, Santa Cruz Biotechnology) and centrifuged. The supernatant and immunoprecipitated proteins were resolved by SDS-PAGE and immunoblotted with anti-RLIP, or anti-Myc antibodies. With Myc-Xreps1(RLIP-BD), the RLIP signal was weakly detected just above an unspecific signal (<). (D) Two µg of GST-Xreps1(253), GST-Xreps1(RLIP-BD), or 1 µg of GST-XralA (G23V) preloaded using GTP-g-S, or GST alone were incubated with Myc-XRLIP (lysate from 10 embryos) and purified with glutathione-agarose beads. The precipitated proteins were resolved by SDS-PAGE, transferred onto a Hybond-P membrane (Amersham) and blotted with a monoclonal anti-Myc antibody (9E10). (E) The table recapitulates the results obtained on the *in vivo* and *in vitro* interaction of XRLIP with Xreps1.

To verify whether the interactions observed using the two-hybrid technique take place *in vitro*, pull-down assays were performed involving Xreps1(253) and XRLIP. Xreps1(253) and XRLIP were synthesized either in bacteria as GST-fusion proteins, or in reticulocyte extracts as Myc-tagged proteins. Specifically, purified recombinant GST-XRLIP was mixed with either reticulocyte-synthesized or embryo-synthesized Myc-Xreps1(253) or Myc-Xreps1(RLIP-BD). The presence of the latter proteins was detected in the GST-pull-down pellets (Fig. 4A, B). The supernatants were almost completely depleted of Myc-Xreps1(253) or Myc-Xreps1(RLIP-BD). No trace of either Myc-Xreps1(253) or Myc-Xreps1(RLIP-BD) could be detected in the pull-down pellets when using purified GST alone. These results show that GST-XRLIP can interact with Myc-Xreps1(253) *in vitro*. Moreover, the interaction between the two partners is sufficiently strong for XRLIP to recover virtually all the available Myc-Xreps1(253).

As XRLIP synthesized in reticulocyte lysates binds Xreps1(253), we explored whether the formation of the XRLIP/Xreps1(253) complex takes place *in vivo* in *Xenopus* embryos. *Xenopus* embryos microinjected with mRNAs coding for Myc-Xreps1(253) or Myc-Xreps1(RLIP-BD) at the 2-cell stage were taken at stage 6–7 (cleavage stage) and expression of the proteins was assessed by Western blot (Fig. 4C). These lysates were used as substrates for co-immunoprecipitation assays and the presence of endogenous wild-type XRLIP was searched in the immunoprecipitation pellets. XRLIP could be very faintly but reproducibly detected in the pellets precipitated *in vivo* only with Myc-Xreps(RLIP-BD) (Fig. 4C).

Since recombinant proteins can be produced in sufficient amounts in the embryo, we tested the capacity of *in vivo*-synthesized XRLIP to interact with Reps1. For this, the GST pull-down technique was used. Specifically, purified recombinant GST-Xreps1(253) or GST-Xreps1(RLIP-BD) was added to cell lysates of *Xenopus* embryos harvested at stage 6–7. A clear but weak Myc-XRLIP band was detected when using 2 µg of either GST-Xreps1(253) or GST-Xreps1(RLIP-BD) (Fig. 4D). To determine whether these results could be observed at others stages of embryonic development or if they were specific of the cleavage stage, similar experiments were conducted with cell lysates from gastrula stage embryos as well as from stage-VI oocytes, and similar results were obtained in all cases (not shown).

To control the efficiency of the pull-down tests, the interaction of Myc-RLIP with GST-XralA G23V, which corresponds to the activated form of RalA, was used. The purified recombinant GST-XralA G23V was preloaded with GTP-g-S and then added to cell lysates of embryos or oocytes at the same stages as described above. Analysis of the pull-down pellets showed that XRLIP was, as expected, present in pellets corresponding to GST-XralA G23V and that the amount of co-precipitated XRLIP did not seem to vary with the stage of the sample. However, as for the endogenous protein, only a small fraction of exogenous Myc-XRLIP was present in these pellets (Fig. 4D). These results summarized in Figure 4E lead to several conclusions. First, the interaction between XRLIP and Xreps1(253) can take place *in vitro* and does not seem to require a third partner. Second, the interaction seems to be more efficient when XRLIP is synthesized in bacteria or yeast. Third, in the embryo only a minor fraction of endogenous XRLIP appears to be available for interaction, whatever the nature of the partner RalA-GST or Xreps). Fourth, the formation of XRLIP-Xreps1(253) complexes during the cleavage stage seems to occur in amounts so low that it almost escapes detection. The previously well-documented interaction between RLIP and GTP-bound Ral takes place in embryos [12]. However, since only a small fraction of endogenous XRLIP seems to be involved in these interactions, we hypothesize that endogenous XRLIP could be in an inert state with regard to Reps. Furthermore, interaction of XRLIP with Reps might be regulated by post-translational modifications.

### Over-expression of Xreps1 during development

**Figure 5:**
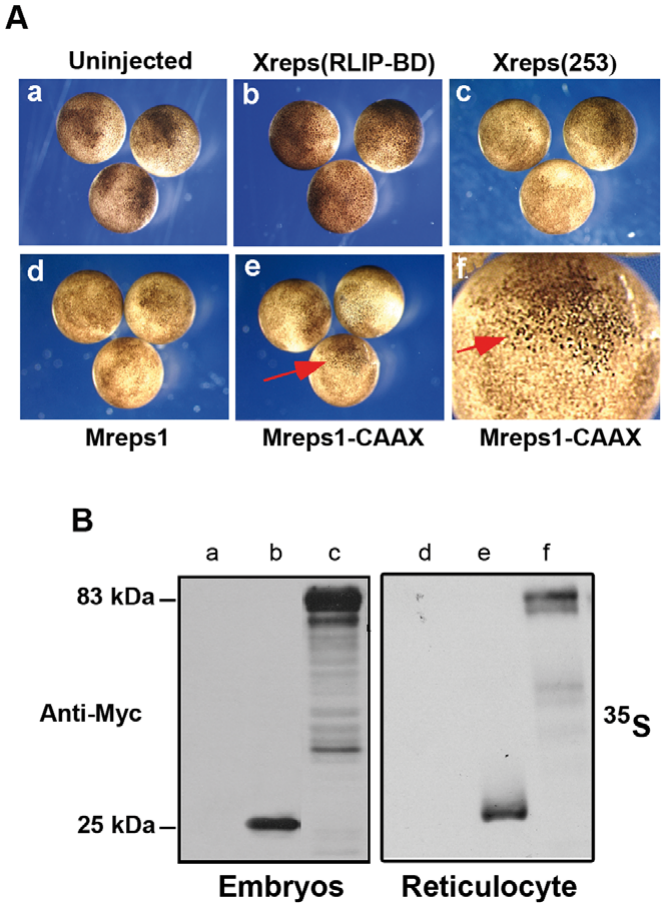
Effect of Xreps1(RLIP-BD) and Xreps1(253) on embryo pigmentation during gastrulation. (A) Animal views of gastrulae. Control uninjected embryos (a). Embryos injected in animal pole of one blastomere at the 2-cell stage with 1 ng mRNA of Myc-Xreps1(RLIP-BD) (b), Myc-Xreps1(253) (c), Mreps1 (d), or Mreps1-CAAX (e) and enlargement of hyperpigmented cell area from an embryo injected with Mreps1-CAAX (f) Red arrows show hypepigmentation area. (B) Protein expression of Xreps1(RLIP-BD) and Xreps1(253) was analyzed by Western blot with a 9E10 anti-Myc antibody, control lanes a, Xreps1(RLIP-BD) b and Xreps1(253) c, and analysis of ^35^S signal from reticulocyte extracts; control d, Xreps1(RLIP-BD) e and Xreps1(253) f.

Xreps1(253) is a *bona fide* partner of XRLIP and is expressed during development. To test whether its over-expression induces a particular phenotype, we microinjected mRNAs coding for the N-terminally Myc-tagged proteins Myc-Xreps1(253) or Myc-Xreps1(RLIP-BD) into the animal pole of one blastomere of embryos at the 2-cell stage. The injected embryos were monitored for visible defects in aspect and/or development throughout embryogenesis up to stage 40. Whatever the amount of mRNA injected, over-expressed Xreps1(253) (up to 3 ng/embryo) caused no visible perturbation of the embryos during early (Fig. 5Ac) or late development (data not shown). Likewise, over-expression of the RLIP-binding peptide Xreps1(RLIP-BD) caused no perturbation (Fig. 5Ab). Similar results were obtained with over-expression of Mreps (Fig. 4Ad). To test if the injected mRNAs were correctly translated, the expression of the exogenous proteins was assessed by Western blot using a monoclonal anti-Myc antibody (Fig. 5B). The two Xreps1 mutant proteins were expressed within hours following microinjection but they did not display the same electrophoretic migration properties. Myc-Xreps1(RLIP-BD) was expressed as a single 25 kDa peptide (Fig. 5B lane b), 12 kDa larger than its theoretical size, as was the case when it was synthesized using reticulocyte extracts (Fig. 5B lane e). The 25 kDa protein was stable and accumulated with time. Myc-Xreps1(253) was expressed as a major 85 kDa product (Fig. 5B lane c), 30 kDa larger than its theoretical size, as was also the case in reticulocyte extracts (Fig. 5B lane f), The 85 kDa protein accumulated with time, but very soon after microinjection several lower-sized Myc-containing peptides could be detected (Fig. 5B lane c). However, these lower-sized Myc-containing peptides did not accumulate in reticulocyte extracts (Fig. 5B lane f), suggesting that in early embryos, Xreps1(253) undergoes either proteolytic processing or partial degradation. In embryos, Xreps1(253) seemed to be translated much more efficiently than Xreps1(RLIP-BD).

Taken together, these data indicate that over-expression of the cloned partial Xreps1(253) or full-length Mreps1 sequences has no obvious effects on embryogenesis. Also intriguing is the lack of effect of over-expression of the RLIP-BD of Xreps1. This result suggests that this peptide might not act as expected by preventing normal interaction between endogenous XRLIP and endogenous Xreps1(253). Another possibility is that Reps might need to be in a particular cellular location to properly function. To test this possibility, and since Reps interacts with RLIP and that RLIP interacts with the RalB protein at the plasma membrane, Mreps1 was fused to a CAAX sequence to target it artificially to the plasma membrane. Mreps1-CAAX was then over-expressed in embryos. Up to MBT, no phenotype could be seen, but later, ectodermal cells became hyperpigmented (Fig. 5Ae and f). This hyperpigmentation persisted throughout development to the tadpole stage (endpoint of the observations made) without inducing developmental failure. At hatching, the hyperpigmented cells became labile and the ectoderm of the tadpole became brittle.

### Effects of mutant RLIP expression on embryo development

The two-hybrid technique showed a clear and unambiguous interaction between Xreps1(253) and XRLIP and was confirmed by our GST-pull down experiments. Yet, over-expression of Xreps1(253) and Mreps1 had no detectable effect on embryo development. However, both proteins, Xreps and XRLIP, might accomplish a function, not clearly observable by a discernible phenotype in our experimental conditions. Moreover, RLIP is a cytoplasmic protein recruited to the plasma membrane by the activated form of Ral. This suggests that Reps could be active only when located in the region of the plasma membrane.

**Figure 6:**
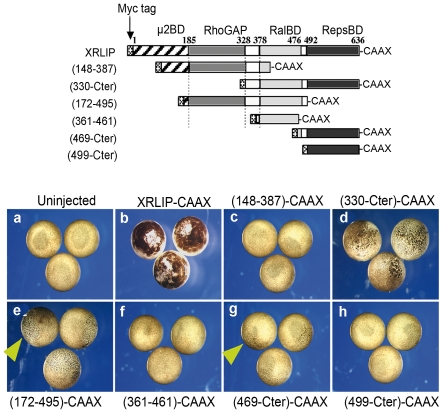
Effect of different mutants of XRLIP mRNAs on embryo pigmentation and cell division. The upper part describes the map of cDNA corresponding to the RNA injected. Lower part (A) uninjected embryos. (B), One blastomere at the 2-cell stage was injected in animal pole with 1 ng of XRLIP-CAAX and the phenotype of the embryos at the cleavage stage is presented. (C–H) Animal views of gastrula embryos injected with the different XRLIP mutants: with (2 ng of (148–387)-CAAX (C) with 1 ng of (330-Cter)-CAAX (D), with 1 ng of (172–495)-CAAX (E), with 2 ng (361–461)-CAAX (F), with 2 ng of (469-Cter)-CAAX (G), and with 2 ng of XRLIP(499-Cter)-CAAX (H). The yellow arrow-heads indicate hyperpigmented cells in the animal hemisphere.

As Mreps1 targeted to the plasma membrane induces a hyperpigmented phenotype, we first tested whether targeting the C-terminal region of RLIP containing Reps-BD induced this phenotype, and then sought to determine the minimal sequence necessary for this interaction. Specifically, embryos were injected at the animal pole with different mRNAs (1 ng/blastomere) coding for deletion mutants of XRLIP and containing a CAAX sequence. None of these mutants caused any visible perturbation during early development corresponding to the cleavage stage. However, as of the MBT, embryos expressing mutant proteins containing the Reps-BD, such as (XRLIP(469-Cter)), presented an altered pigmentation (Fig. 6D, E and G). This altered pigmentation was similar to the phenotype induced by Xreps1-CAAX (Fig. 5Af) but quite different from that previously observed with XRLIP-CAAX (Fig. 6B) [11]. These embryos developed almost normally, often reaching stage 17 (late neural stage) (71%+/−12 n = 169) and even later stages. The ectoderm composed of hyperpigmented cells was more fragile and usually broke at later stages, most often causing the death of the embryos. Clusters of hyperpigmented cells often detached from the embryo and remained linked to the vitelline membrane after hatching. Embryos injected with XRLIP(172–495)-CAAX mRNA, displayed the same dotted aspect (Fig. 6E), while XRLIP (499-Cter)-CAAX induced only very weak hyperpigmentation. This would seem to rule out the implication of Reps1 in the phenotype described, but it should be stressed that only the XRLIP(172–495)-CAAX mutant still contained the 14 residues of the N-terminus of RepsBD, shown to mediate the interaction with Xreps1. Other XRLIP deletion mutants containing the interacting domain with Reps1 as well as full-length Mreps1, both targeted to the membrane, induced similar hyperpigmentation phenotypes (Fig. 5Ae and Fig. 6E and G). These results suggest that RLIP and Reps1 could interact at the plasma membrane to induce the hyperpigmentation phenotype.

### Rescue of mutant phenotypes by expression of Xreps1(RLIP-BD) and synergistic effect of Xreps1(253)

**Figure 7:**
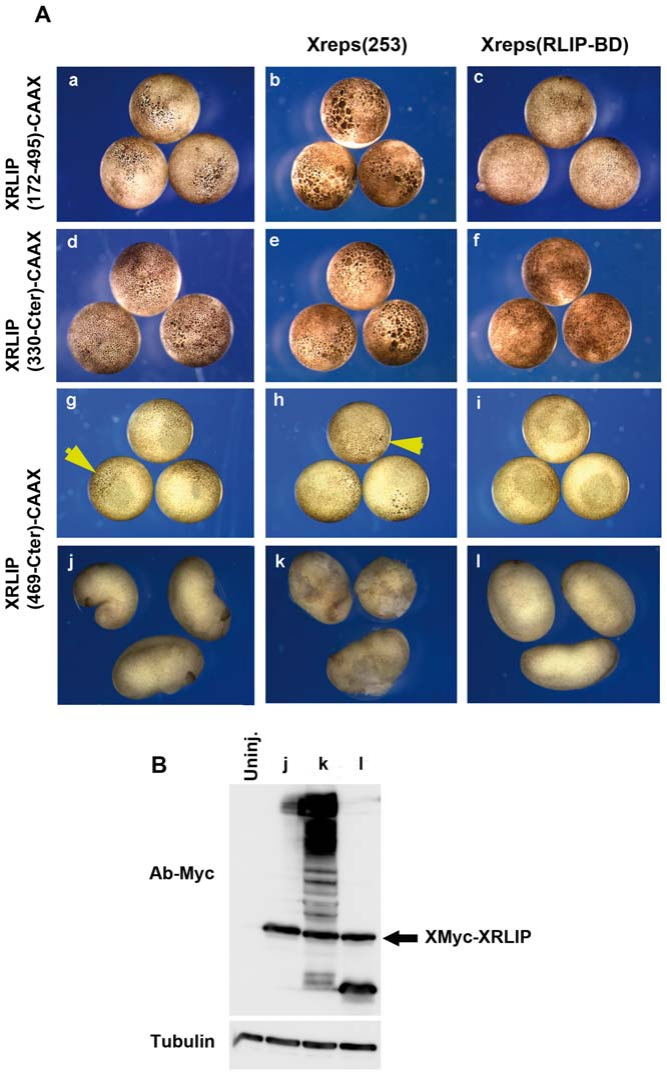
Phenotypes induced by XRLIP(172–495)-CAAX, XRLIP(330-Cter)-CAAX or XRLIP(469-Cter)-CAAX are amplified by Xreps1(253) and are rescued by Xreps1(RLIP-BD) but not. (A) The injected embryos were photographed at the gastrula stage. Embryos at the 2-cell stage were injected (a, d, g) with 1 ng of respectively XRLIP(172–495)-CAAX, XRLIP(330-Cter)-CAAX or XRLIP(469-Cter)-CAAX mRNA alone, or co-injected (b, e, h) with 1 ng of Xreps1(253) mRNA, or co-injected (c, f, i) with 1 ng of Xreps1(RLIP-BD) mRNA. Only the hyperpigmented phenotype (a, d, g) disappears (c, f, i). In g and h the yellow arrows show the hyperpigmented cells. (B) Proteins expression, from embryos injected with XRLIP(469-Cter)-CAAX alone (j) or respectively co-injected with Xreps (k) and Xreps(RLIP-BD) (l), were analyzed by Western blot with a 9E10 anti-Myc antibody.

The hyperpigmentation induced by the over-expression of the XRLIP mutants ((XRLIP(469-Cter)-CAAX, XRLIP(330-Cter)-CAAX, XRLIP(172–495)-CAAX) and Mreps-CAAX) suggests that Xreps1 and XRLIP interact close to the plasma membrane to generate the phenotype. To investigate this possibility, we tested by co-injection whether the Xreps1(RLIP-BD) mRNA could compete the endogenous Xreps and rescue the phenotype induced by the expression of the proteins XRLIP(330-Cter)-CAAX, XRLIP(172–495)-CAAX, or XRLIP(469-Cter)-CAAX. Interestingly, the hyperpigmented phenotypes induced by XRLIP(172–495)-CAAX (Fig. 7Aa), XRLIP(330-Cter)-CAAX (Fig. 7Ad), or by XRLIP(469-Cter)-CAAX (Fig. 7Ag), were almost completely rescued when Myc-Xreps1(RLIP-BD) was co-injected (Fig. 7Ac, Af and Ai). Oversized blastomeres were no longer observed and pigmentation of animal blastomeres was practically undistinguishable from that of uninjected embryos. To test whether Xreps1(253) could synergize the effect of the XRLIP-CAAX deletion mutants (Fig. 7Aa, d, and g) by itself and not by competing with another protein, mRNAs coding for these three mutants were co-injected with mRNA coding for Xreps1(253) (Fig. 7Ab, e, h and k). The Xreps1(253) sequence weakly amplified the effect of these three mutants. When XRLIP(172–495)-CAAX, XRLIP(330-Cter)-CAAX or by XRLIP(469-Cter)-CAAX were injected at doses unable to cause any visible alteration, co-injection with large amounts of Xreps1(253) led the appearance of small dotted patches (Fig. 7 Ab, e and h) characteristic of the phenotype obtained at higher doses (Fig. 5Ad). Over-expression of XRLIP(469-Cter)-CAAX induced a weak effect on ectodermal cells of embryos, but not only was this effect synergistically increased in the presence of Xreps1(253), but at later stages of development, during the tailbud stage, the embryo morphology was affected and ectodermal cells were detached from the ectoderm (Fig. 7 Ak). To test whether Xreps1(RLIP-BD) could rescue all the effects of XRLIP-CAAX, mRNAs coding for full-length XRLIP-CAAX and for Xreps1(RLIP-BD) were co-injected (not shown). Xreps1(RLIP-BD) did not alter the depigmentation induced by XRLIP-CAAX (not shown). This indicates that Xreps1(RLIP-BD) specifically blocks the action of the XRLIP mutants containing the Reps-BD sequence. Hence, Xreps1(253) participates in pathways relying on interactions involving the C-terminal part of XRLIP-CAAX. Moreover, the blocking action of Xreps1(RLIP-BD) is probably not due to the masking of closely positioned domains necessary for recruitment of another target protein, and the 513 N-terminal amino acids of Reps1 are probably sufficient to induce hyperpigmentation and to arrest blastomere division.

### XRLIP-CAAX translocates Xreps1 to the plasma membrane by its C-terminal domain

**Figure 8:**
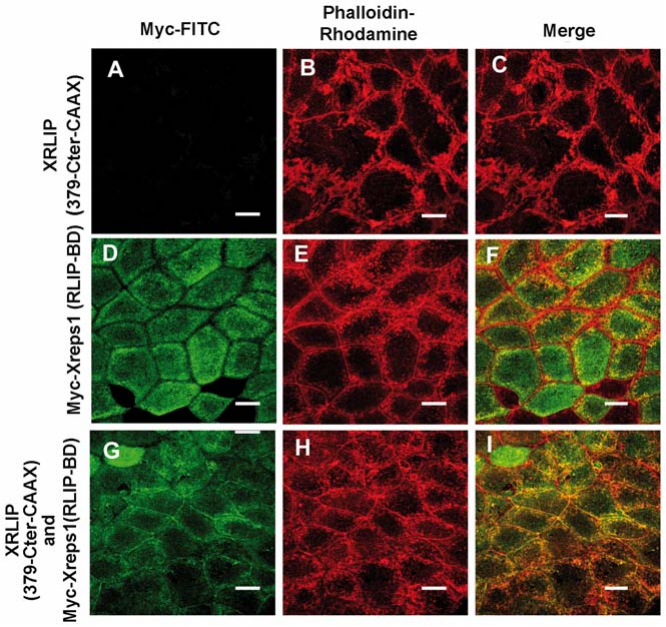
Xreps1 protein interacts with XRLIP-CAAX at the plasma membrane. Representative confocal micrographs of animal caps dissected at the 2000-cell stage, showing the distribution of Myc-Xreps1(RLIP-BD)-CAAX expressed alone (A) or in the presence of XRLIP(379-Cter)-CAAX (G). Myc-Xreps1(RLIP-BD) mRNA (1 ng) and XRLIP(379-Cter)-CAAX (0.5 ng) were microinjected into the animal hemisphere of 2-cell stage embryos and Myc-Xreps1(RLIP-BD) was visualized with an FITC anti-Myc antibody. The animal caps were fixed and Myc-XRLIP(379-Cter)-CAAX and Myc-Xreps1(RLIP-BD) immuno-stained with an FITC anti-Myc antibody, (panels A, D and G stained in green), the F-actin with phalloidin-rhodamine, (panels B, E and H stained in red) and the merge of Myc-FITC with phalloidin-rhodamine are shown (panels C, F and I). Scale bars represent 50 µm.

Since biochemical analyses and rescue experiments show that *in vivo* Xreps1 interacts weakly with endogenous and exogenous XRLIP, we investigated whether this interaction was visible in embryos after the MBT stage. Using confocal microscopy, the distribution of Xreps1 expressed alone or with the deletion mutant of XRLIP-CAAX coding for the C-terminal part was examined. Animal caps dissected from embryos injected with Xreps1(253) (not shown) or Xreps1(RLIP-BD) (Fig. 8D) revealed that the localization pattern of the protein was strikingly similar to that of XRLIP [12]. In some blastomeres both proteins were diffuse throughout the cell, whereas in others, they were excluded from the central zone. In other cells Xreps1(253) and Xreps1(RLIP-BD) localized towards the cell periphery and in their central part, but the proteins were excluded from a pericentral zone. However, in all cases they were totally excluded from a zone that seems to correspond to the cell cortex. When untagged XRLIP(379-Cter)-CAAX mRNA was co-injected with Myc-tagged Xreps1(RLIP-BD), the cortical actin of the animal blastomeres was not different from that of controls (Fig. 8H). This result is identical to that obtained with XRLIP(330-Cter)-CAAX, previously described. But, as this mutant contains a sequence that induces the formation of large ectodermal cells, due in part to the presence of a sequence that affects cytokinesis (manuscript in preparation), we preferred, in this experiment to use an mRNA encoding a protein lacking the amino-acid sequence involved in this additional phenotype. Nevertheless, there was clear partial localization of Xreps1(RLIP-BD) at the plasma membrane (Fig. 8G) of the blastomeres and co-localization with cortical actin, situations never observed when Xreps1(RLIP-BD) was injected alone (Fig. 8F). This further confirms that the interaction between XRLIP and Xreps1(253) occurs in embryos, and that when XRLIP is located in the plasma membrane it can recruit Xreps1(253).

## Discussion

Using a two-hybrid screening approach, several putative partners of XRLIP expressed during early development were isolated. One of them is the *Xenopus* ortholog of Reps1. In agreement with what had previously been shown [15], interaction between Reps1 and RLIP is specific and is evolutionarily conserved. Using GST pull-down and co-immunoprecipitation assays, we confirmed that this interaction in *Xenopus* is regulated during early development. Moreover, the interacting domain of each partner was redefined. Previously [15], the Reps-BD of RLIP76 was defined by the last 476 amino acids of the protein. It is shown here that the 78 C-terminal amino acids of Xreps1 are necessary and sufficient to bind XRLIP. These results support a model in which the coiled-coil sequence of Reps1 participates in binding to RLIP. However, this coiled-coil sequence is not composed of perfect L(6×)L repeats and it has been proposed that the coiled-coil region of Reps1 alone is not sufficient to interact with RLIP [15]. The C-termini of Reps1 and POB1/Reps2 also have predicted coiled-coil regions, and their alignment shows that the last 70 C-terminal residues are 86% identical. Thus, since the coiled-coil region of Reps1 alone, which comprises 50% of this domain, does not interact with RLIP, the RLIP binding region could be located between residues 475 and 492 at the C-terminus, or might only partially overlap the predicted coiled-coil domain. This C-terminal part of RLIP involved in the production of a phenotype during early development can be divided into two distinct domains, one defined by the segment 330–476, the expression of which affects cell division (unpublished results), and a more restricted domain encompassing the segment 475–492 that interacts with Reps1 and induces hyperpigmentation of cells later in development.

Over-expression in embryos of the most N-terminal 513 amino acids of Xreps1(253) or of the full-length Mreps1, does not affect development in any obvious way. Xreps1(RLIP-BD) but not Xreps1(253) can suppress the phenotype induced by over-expression of the XRLIP truncated versions (330-Cter), (172–495) or (469-Cter) containing the CAAX sequence. Consequently, these proteins interact, and Xreps1 or at least its 513 C-terminal amino acids are required for the phenotype to appear. Furthermore, the region of XRLIP corresponding to amino acids 475 to 492 is necessary for the interaction with Xreps1(253) and is involved in the XRLIP-induced phenotype. Indeed, when XRLIP(330-Cter)-CAAX or XRLIP(172–495)-CAAX are coinjected with Xreps1(RLIP-BD) they increase the effect of XRLIP-CAAX on pigmentation, while their co-injection with Xreps1(RLIP-BD) rescues or almost completely suppresses the hyperpigmentation phenotype. Moreover, co-injection of XReps1(253) with these truncated mutants of XRLIP partially restores the hyperpigmentation and cell division phenotypes. Consequently, Xreps1 is required to establish these phenotypes and the N-terminal part not encoded by the partial cDNA XReps1(253) of Xreps1 is not necessary for this effect. Dominant positive XRLIP-CAAX deletion mutants, containing the 476 C-terminal amino acids of XRLIP such as (330-Cter), (469-Cter), recruit proteins such as Xreps1(253) that might participate in establishing an extensive array of protein interactions. However, the effect of the mutant XRLIP(330-Cter)-CAAX on cell division cannot be due to the action of Xreps1 because XRLIP(469-Cter) induces only the hyperpigmentation phenotype. This conclusion is confirmed by the phenotype induced by the expression of a deletion mutant containing only the sequence 330–476, for which only cytokinesis is disturbed (manuscript in preparation).

Injection of a deletion mutant of XRLIP(172–495)-CAAX composed of RhoGAP domain and the RalBD can produce the same effects as XRLIP(330-Cter)-CAAX. As mentioned above, the subcloned domains of XRLIP slightly overlap to some extent. Thus, the XRLIP(172–495)-CAAX mutant overlaps the first 26 residues of XRLIP(469-Cter) as defined by the group of Feig [15]. Indeed, interaction with Xreps1(253) is possible as shown by the two-hybrid technique. This result is at least partly confirmed by the absence of interaction in the two-hybrid system of Xreps1(253) with the XRLIPΔμ2 (Δ 475–492) mutant corresponding to the deletion of the overlapping region between the RalBD and Reps domains.

Xreps1 cannot suppress the phenotype induced by full-length XRLIP-CAAX, even though this mutant possesses an intact Xreps1(RLIP-BD). Thus, Reps1 would be a component of a different pathway not involved in the early phenotype as shown by the absence of phenotype when Xreps1(RLIP-BD) is injected. The disrupted early pathway would lead to remodeling of the F-actin cytoskeleton, since cortical actin disruption is observed only when XralB G23V, XRLIP-CAAX or C-terminally truncated XRLIP-CAAX mutants are over-expressed. The late pathway would involve Reps1 and have an impact on different targets not leading to the perturbation of the F-actin cytoskeleton, since cortical actin seems virtually untouched in XRLIP(330-Cter)-CAAX injected embryos.

The confocal microscopy analysis of the cellular location of XRLIP suggests a dynamic pattern. XRLIP appears to be located at first throughout the cell. It is then gradually excluded from a central (perinuclear) zone that expands towards the periphery of the cell. The localization pattern of XRLIP and that of Xreps1(253) are strikingly similar. Either each protein is positioned by independent mechanisms, or the two proteins are located in the same complex. However, biochemical evidence argues against the latter possibility because no significant amount of the XRLIP/Xreps1(253) complex was detected *in vivo*, at least during the pre-MBT stages (unless the interaction is very dynamic, i.e. low affinity constant for the *in vivo* interaction).

Within the limits of sensitivity of the pull-down technique and before the MBT stage, endogenous XRLIP does not seem to interact with Xreps1(253). The XRLIP used for co-immunoprecipitation was unmodified in its C-terminal amino acids, but for other analyses such as phenotypic tests and confocal microscopy, XRLIP was fused to CAAX. Therefore, in the cytosol, XRLIP might undergo post-translational modifications modulating its interaction with Xreps1. Interestingly, targeting of XRLIP to the plasma membrane might restore accessibility of the protein to the Reps1 binding site or increase the interaction between these two proteins.

**Figure 9:**
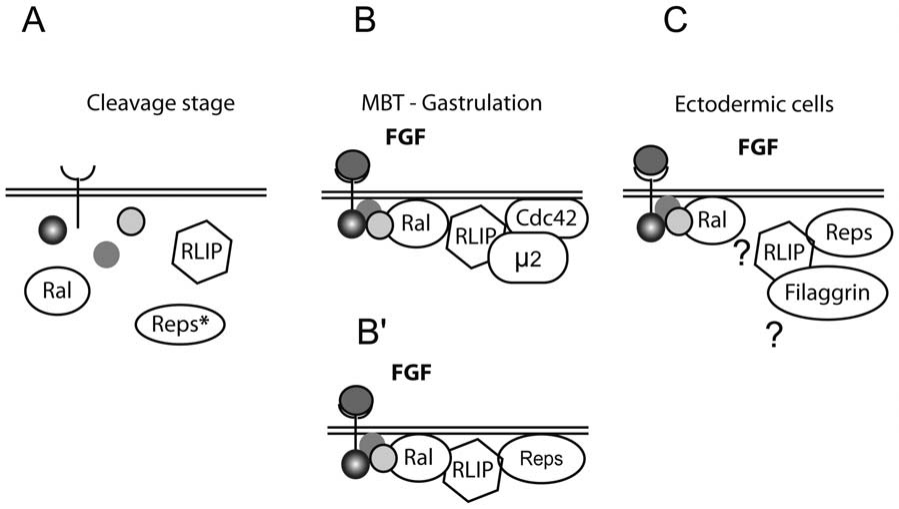
Hypothetical model of the interaction of Reps with RLIP during early development. (A) During cleavage stage, and up to MBT, Ral, RLIP and Reps are present in embryo; however no interactions are detected between them. (B) As of MBT, Ral interacts with RLIP and recruits it at the plasma membrane. It is thought that through Cdc42 it destabilizes the actin cytoskeleton and through μ2 and consequently AP2 allows endocytosis via clathrin. At this stage of development RLIP interacts also with Reps1, but (C) this interaction could be exclusive of ectodermic cells and could use another partner, such as filaggrin.

The nature of the targets of Reps1 remains speculative. Based on the data of the structurally related protein POB1/Reps2, Reps1 might interact with essential proteins of the receptor-mediated endocytotic pathway. It seems unlikely that a mechanism such as receptor-mediated endocytosis plays a role in early pre-MBT development that is characterized by a succession of mitoses. Moreover, the first manifestation of signaling activity, defined by the expression of the earliest gene does not occur before stage 8. Additionally, endocytosis would be less active during mitosis [32], since it has been shown that many endocytotic proteins are phosphorylated during mitosis so as to down-regulate this process. Indeed, the Epsin/Eps15/POB1 complex is disrupted by phosphorylation. The group of Camonis [33] has shown that RLIP facilitates phosphorylation of Epsin, a target of Reps1, by cdk1, reinforcing the possibility that RLIP and Reps1 would not interact before the establishment of a complete cell cycle with G1 and G2 phases at the MBT stage. To explore a putative protein interaction network with RLIP, liquid chromatography/electrospray ionization tandem mass spectrometry was used. Among the groups of genes forming different complexes interacting with RLIP, filaggrin, has been identified through a peptide of 13 amino acids and the analysis yields a score of 43 with the “mascot” program. Other groups of peptides have been selected for their interactions with RLIP with scores above 30, such as 7 peptides coding for Reps1 with scores between 30 and 62, one peptide of 10 amino acids coding for POB1/Reps2 with a score of 41, and 6 peptides of 12 amino acids coding for RalA with scores comprised between 34 and 54. During the cleavage stage, up to MBT, Ral, RLIP and Reps are present in embryo (Fig. 9A), but no interaction is detected. However, from MBT, Ral interacts with RLIP and recruits it at the plasma membrane (Fig. 9B). It has been proposed that through Cdc42 RLIP destabilizes actin cytoskeleton and through its interaction with μ2 of AP2 complex it allows endocytosis at clathrin. At this stage of development RLIP interacts also with Reps1 (Fig. 9B′), but this interaction could be exclusive of ectodermic cells and could use another partner, the filaggrin (Fig. 9C). Filaggrin is known to be associated with skin barrier abnormalities [34] and its deficiency leads to a leaky skin barrier [35]. This function of filaggrin can be related to our observation of loss of ectodermal cells when XRLIP-CAAX and Xreps1(253) are co-overexpressed. In mice, the skin barrier appears relatively late in development at stage (E16) [36]. In *Xenopus*, the bilayer epithelium ectoderm is generated during the blastula stage [37] before hatching, and could explain why desquamation of embryos before hatching was observed (Fig. 7 Ak). Since no specific spatial expression of Reps1 was detected in embryos, Reps1, similary to RLIP or some effectors of FGF signaling pathway, is not active by itself, but through its interaction with RLIP. Such interaction might be controlled by a post-translational modification of RLIP or Reps1 itself.

In summary, our results indicate that the 78 C-terminal residues of Xreps1 interact with the 475 last amino-acids in C-terminal part of XRLIP. This interaction is not detected during the cleavage stage, which is defined by active mitotic activity, but only from gastrulation. Differential formation of the RLIP and Reps1 complex indicates that this interaction is not constitutive but that it is developmental-stage specific. The underlying mechanisms controlling the formation of this complex remains to be described. Moreover, the persistent defect specifically induced by Reps-CAAX in ectodermic cells of embryos could suggest that Reps is functional in ectodermic cells.

## Materials and Methods

### Ethics statement

Our work uses early *Xenopus* embryos. All experimental procedures described in this study followed the recommendations of the “Comité National de Réflexion Ethique sur l'Experimentation Animale” of the Ministry of Higher Education and Research and were approved by the “Comité d'Ethique Buffon” license N° CBE-009-2011, covering all the ethical aspects. This work required no other license.

### Cloning

All cDNAs used for microinjection purposes were subcloned into the vector pRN3 [24] that contains the 5′ and 3′ untranslated regions of the *Xenopus* globin gene and a poly(A) tail. Plasmids were linearized with the restriction enzyme Asp718 prior to RNA synthesis.

The various XRLIP mutants, namely XRLIP(469-Cter), XRLIP(172–495), XRLIP(330–Cter), XRLIP-CAAX and the Xreps1 mutants Xreps1(RLIP-BD) and Xreps1(ΔRLIP-BD), were PCR-amplified using primers containing convenient restriction sites and encoding or not the XralB-CAAX motif, and were subcloned into pRN3 containing two in frame Myc-Tags. All the constructs were verified by sequencing to exclude the presence of PCR-induced mutations.

For the two-hybrid interaction tests all XRLIP mutants without the CAAX motif were subcloned into pNLX3. Xreps1(253), Xreps1(RLIP-BD), and Xreps1(ΔRLIP-BD) were subcloned into pGAD-GE. RLIP76 cloned into pNLX3 was a kind gift of J. Camonis.

For the GST pull-down experiments, wild-type XRLIP, Xreps1 and Xreps1(RLIP-BD) were subcloned into pGEX-4T-1 (Pharmacia). The clone pGEX-RalA G23V was kindly supplied by J. Camonis.

### Yeast two-hybrid screen

The yeast strains L40 and AMR70 were kindly provided by J. Camonis. The host yeast strain was L40 (MATa, trp1, leu2, his3, LYS2::lexA-HIS3, URA::lexA-lacZ). RLIP76 cDNA cloned in the vector pNLX3 was used as bait for library screening. This construct codes for the human RLIP fused at is C-terminus to the DNA-binding domain of the LexA protein, previously modified to contain a nuclear localization signal. The *Xenopus* oocyte cDNA library was constructed using the vector pGAD-GE [25]. The two-hybrid screen was carried out as previously described [25].

For interaction specificity studies, one of the two following methods was used: i) the L40 strain containing the pGAD-based constructs was mated with the AMR70 strain (MATa, trp1, leu2, his3, LYS2::lexA-HIS3, URA::lexA-lacZ) containing the pNLX3-based constructs, or ii) the L40 strain was co-transformed with pairs of pNLX- and pGAD-based constructs. Diploids or co-transformants were tested for His^+^ (auxotrophy) and LacZ^+^ expression, and growth in selective medium.

### mRNA synthesis and embryo microinjection

*Xenopus* were purchased from the CNRS frog colony (Rennes, France). Embryos were fertilized *in vitro* and dejellied with 2% cysteine-HCl in 0.3× modified Barth's solution at pH 7.6 (MBS) [26], and incubated in 1× MBS. Capped mRNAs were synthesized using the T3 mMessage mMachine System Kit (Ambion) and the mRNAs were subjected to sequential precipitation with 0.5 M ammonium acetate followed by 2.5 M LiCl to remove unincorporated nucleotides. Microinjections, using a Drummond microinjector, were performed at the two-cell stage in the animal pole in 1× MBS with 2% Ficoll unless stated otherwise. Embryos were transferred to 0.1× MBS with 2% Ficoll, 4 h after injection, and incubated at 16–18°C until the desired stage was reached.

### Western blots

Embryos were harvested at stage 6–7, according to Nieuwkoop and Faber [27], and lyzed in 40 µl of buffer D (50 mM Tris-HCl pH 7.5, 100 mM NaCl, 10 mM MgCl_2_, 1% Triton X-100, 1 mM PMSF) per embryo by pipetting up-and-down several times, then treated twice with the same volume of Freon (Merck) to remove the yolk [28]. The crude protein extracts were mixed with Laemmli buffer and subjected to SDS-PAGE. The proteins were then transferred to PVDF membranes (Hybond-P, Amersham) and probed overnight with the appropriate antibodies (Santa Cruz Biotechnology). Signals were detected by chemioluminescence (ECL, Amersham) after incubation with peroxidase-conjugated secondary antibodies (Sigma).

### In vitro protein synthesis

Myc-XRLIP, Myc-Xreps1(253), Myc-Xreps1(RLIP-BD), and XralB G23V proteins were synthesized using the reticulocyte extract TnT Kit (Promega) and labelled with [^35^S] methionine (ICN) according to manufacturer's instructions.

### Whole-mount in situ hybridization

Whole-mount *in situ* hybridization was performed as described [29] with reps1 and with chordin as control. After satisfactory color development, embryos were fixed in MEMFA (0.1× M MOPS pH 7.4, 2 mM ethylene diamine tetra-acetic acid and 3.7% formaldehyde) for 1 h at room temperature, washed, and stored in 100% ethanol. Embryos were also treated in 10% H_2_O_2_ to bleach the pigment.

### Synthesis and purification of the GST-fusion proteins

cDNAs coding for XRLIP, Xreps1(253) and Xreps1(RLIP-BD) were cloned fused to the open reading frame of GST in the pGEX-4T-1 vector. Protein synthesis was induced by 1 mM IPTG (Euromedex). Bacterial clones containing GST-RalA G23V were subsequently incubated at 37°C, whereas clones containing GST-XRLIP, GST-Xreps1(253) and GST-Xreps1(RLIP-BD) were incubated at 30°C to limit protein degradation. When the appropriate incubation time was reached, the cultures were centrifuged and the bacterial pellets were resuspended in buffer D. Cell lysis was achieved by adding 1 mg/ml lysozyme (Euromedex), and after incubation for 1 h at 4°C, the samples were sonicated and centrifuged at 4°C for 45 min at 14000 rpm, to remove cell debris and insoluble material. The supernatants were immediately used.

Recombinant GST-proteins were purified on glutathione Sepharose 4B (Amersham) at room temperature in buffer D as described by the manufacturer. The purified products were analyzed by SDS-PAGE and the concentration of the purified recombinant GST-proteins was estimated by Coomassie brilliant blue staining using a BSA standard.

### GST pull-down assays

GST pull-down assays were carried out using 2 µg of GST-XRLIP, GST-Xreps1(253) or GST-Xreps1(RLIP-BD). Controls were carried out with 5 µg of GST-RalA G23V. This small G protein was preloaded with GTP-g-S in Mg-free loading buffer (20 mM Tris-HCl pH 7.5, 25 mM NaCl, 10 mM EDTA, 1 mM DTT, 0.05% BSA and 1 mM GTP-g-S) for 1 h at 37°C prior to use. Addition of 20 mM MgCl_2_ stopped the reaction. Using this method, the yield of GTP loading was estimated to be 30%. The embryos were lyzed in buffer D and treated twice with Freon. Reticulocyte extracts were diluted in buffer D. Crude protein extracts were pre-incubated with 5 µg of GST alone in buffer D for 1 h at room temperature and centrifuged. The supernatants were then incubated with the corresponding GST-fusion protein for 2–3 h at room temperature and centrifuged. All pellets were washed 3 times with ice-cold buffer D and resuspended in Laemmli buffer prior to Western blot analysis.

### Co-immunoprecipitation assays

Embryos were injected with mRNA coding for the appropriate Myc-tagged protein. At the 2000 cell stage, proteins were extracted by lysis of 15 to 20 embryos in 800 µl of buffer (50 mM Tris-HCl pH 7.5, 100 mM NaCl and 2 mM PMSF). After incubation for 15 min in ice, the extracts were centrifuged at 13,000 rpm for 30 min. The supernatants were incubated with slow shaking at 4° in the presence of 3 µl of anti-myc monoclonal antibody (anti c-Myc (9E10), Santa Cruz) already bound to agarose and centrifuged at 13,000 rpm for 6 min. The supernatant was discarded and 32 µl of 1× Laemmli buffer were added to the pellet. The precipitated proteins were separated by SDS-PAGE and transferred onto a PVDF membrane for Western blotting.

### Confocal microscopy

The vitelline membrane of selected embryos was removed manually and the embryos were permeabilized for 15 min at room temperature in 80 mM Hepes, pH 6.8, 1 mM MgCl_2_, 100 mM EDTA, 30% glycerol and 0.1% Triton X-100 and fixed in 1× PBS and 3.5% formaldehyde, for 1–2 h at room temperature. The embryos were then incubated in saturation buffer (1× PBS, 1% BSA) for 5 h at room temperature under mild shaking. Immunostaining was carried out on explants with c-Myc antibody (9E10) (dilution 1∶1000 in 1× PBS, 1% BSA and 0.1% Triton X-100), followed by FITC-conjugated secondary antibodies (Jackson Immuno-research). Animal caps were manually dissected and treated with rhodamine-phalloidin (7.5 µg/ml (Cytoskeleton)). They were washed in 1× PBS and 0.1% Triton X-100, and mounted in Citifluor medium. Observations were carried out with a Leica TCS-4D laser confocal microscope (Leica Instruments).
